# Access to technology and foundational math proficiency among students: empirical evidence from India

**DOI:** 10.1057/s41599-025-05224-w

**Published:** 2025-07-04

**Authors:** Prashant Poddar, Valentina Rotondi, Ridhi Kashyap

**Affiliations:** 1 Department of Sociology, Nuffield College, and Leverhulme Centre for Demographic Science, University of Oxford, Oxford, UK; 2 Department of Business Economics, Health and Social Care, University of Applied Sciences and Arts of Southern Switzerland (SUPSI), Lugano, Switzerland

**Keywords:** Development studies, Economics, Education, Science, technology and society

## Abstract

Digital resources such as laptops have the potential to improve access to educational resources and provide personalized and uninterrupted learning opportunities for students. The impacts of these technologies may be especially salient in contexts where classroom sizes are large and schooling quality is poor. Here, we study the impacts of access to laptops on foundational math proficiency in one such context, i.e., India, exploiting plausibly exogenous variation in the implementation of the Tamil Nadu Free Laptop Scheme (TFLS). Introduced in 2011, the TFLS was one of the largest and targeted free laptop programs in the world, distributing over 5 million laptops. Using data from the Annual Status of Education Report (ASER) and India Human Development Survey (IHDS) within a double difference design, we show positive effects of access to laptops on foundational math proficiency of students, with the largest improvements experienced by those in economically disadvantaged households. We further find that these positive effects on foundational math proficiency are complemented by improvements in other education-related outcomes of students, such as more time spent on learning, better comprehension of language, and a reduction in private tuition. We provide evidence that laptops are able to close economic and gender divides in education. Our results remain robust to a variety of falsification and sensitivity checks.

## Introduction

The United Nations’ Sustainable Development Goal 4 (SDG 4) emphasizes the need for quality education for children.^1^ With the digitalization of educational resources, access to technologies, such as laptops and computers, constitutes an important part of the education infrastructure necessary to achieve SDG 4. Digital technologies not only help to provide a wide variety of learning resources to students but also enable them to continue their learning outside of traditional classroom settings, which in low- and middle-income country (LMIC) contexts can often be crowded, difficult to reach, or inaccessible (Adukia, 2017; Dhawan, 2020; Fuller, 1985; Hanushek, 2013; Masino and Niño-Zarazúa, 2016; Orazem and King, 2007). Technologies such as laptops may in these settings provide opportunities for more focused and private learning than may be possible in classroom settings. Digital technologies become especially important during times of crises, such as the COVID-19 pandemic, that can result in learning losses for students and potentially increase educational inequalities (Aucejo et al., 2020; Bacher-Hicks et al., 2021; Engzell et al., 2021; Grewenig et al., 2021). Access to devices such as computers and laptops during these times can help students to continue their learning and minimize educational losses (Clark et al., 2021). Digital devices also have the potential to help adolescents acquire digital skills that are useful when they enter increasingly digitalized labor markets.

Despite the plausible benefits from digital resources, progress in terms of the availability of computers, laptops, and other digital technologies remains uneven in different parts of the world. While there is near-universal access to computers in upper secondary schools in high-income countries, low-income countries still lag behind.^2^ In an effort to close these gaps, different laptop distribution programs have been implemented across LMICs. Although the exact features, extent of coverage, and investment in these programs have varied across settings, the objectives of these policies have been to improve access to laptops among children of school-going age. However, considerable differences still persist, with data suggesting that in 2019, only 38.5% of households had a computer at home in developing countries when compared to 82.3% of households in developed ones (International Telecommunication Union, 2019).

In this study, we explore the effects of laptop accessibility on upper secondary students’ foundational math skills in India, specifically delving into the Tamil Nadu Free Laptop Scheme (TFLS). The state-level initiative introduced in 2011 distributed over 5 million complimentary laptops to students attending government and government-funded institutions between 2011 and 2020.^3^ By capitalizing on potentially exogenous variation in the program’s execution, our aim is to discern whether the availability of laptops influenced upper secondary students’ proficiency in foundational math skills. India provides an important context to study the impact of digital resources on foundational skills because even as enrollment rates have been high in recent years, the learning level among students remain low (Beg et al., 2023; Chatterjee and Poddar, 2021; Das and Zajonc, 2010; Muralidharan et al., 2019). According to recent data from the Annual Status of Education Report (ASER), the enrollment rate among children aged 14–18 was 86.8% in the year 2023. Over 50% of students in this age group, however, did not possess basic math skills like simple division that is generally expected from a third or fourth standard (grade) student (ASER Center, 2024). Research suggests that foundational skills in math and numeracy are important determinants of labor market opportunities (Charette and Meng, 1998; Cherry et al., 2020), and the returns for basic skills in Math and English are high in the manufacturing sector as well as for semi-skilled work (Amin et al., 1998; Heath and Mobarak, 2015; Pāla-Majumadāra and Begum, 2006; Wedege, 2002).

Existing literature on the impact of digital resources such as laptops and computers on the academic proficiency of students largely points towards mixed results (Murnane and Ganimian, 2014). For instance, Beuermann et al. (2015) and Cristia et al. (2017) evaluated the impact of the influential One Laptop Per Child (OLPC) program. OLPC is a non-profit organization that started at MIT in 2005 and attempted to improve the availability of laptops for school-going children around the world. The researchers studied the program in the context of Peru and found little to no effects of access to laptops on test scores and cognitive skills of primary school students. Mora et al. (2018) studied the impact of the eduCAT program that provided laptops to students coupled with wireless connectivity in the context of Catalonia and found negative impacts of the program on language and math skills of secondary school students, with the negative impact being larger for boys than for girls. Mo et al. (2013), however, found positive effects of providing laptops bundled with remedial tutoring software on the math scores of students in China. In the context of home computers, Malamud and Pop-Eleches (2011) studied their impacts in Romania and found negative impacts on grades but positive impacts on the cognitive skills of students. Fairlie and Robinson (2013) also found no impacts of home computer ownership on educational outcomes of students in the United States. Furthermore, Fairlie (2016) found no differential impact of home computers on learning outcomes of boys relative to those of girls. Hall et al. (2021) found that access to digital resources such as laptops can even worsen socioeconomic inequalities in learning.

Our study contributes to the wider literature on the impact of digital resources such as laptops or computers on the educational outcomes of students, but is unique in three specific aspects. First, most of the research on the subject has focused on the causal impacts of laptops and computing resources on educational outcomes of primary and middle school students (Beuermann et al., 2015; Bulman and Fairlie, 2016; Cristia et al., 2017). Our study, on the other hand, studies these impacts on the learning outcomes of upper secondary students. This is an important differentiating factor as upper secondary students are much closer to entering the labor market, and any effects of the program on foundational math skills of students at this stage can have potentially positive labor market outcomes later on (Heath and Mobarak, 2015; Pāla-Majumadāra and Begum, 2006). Moreover, impacts on educational performance and skills development at this stage of the educational trajectory can have more immediate, short-term implications for access to post-secondary education and/or job training opportunities.

Secondly, our study sheds light on novel mechanisms by which laptop accessibility can enhance educational outcomes. While we show suggestive evidence for higher ownership of laptops amongst households with *treated* students, we also find significant changes in “first-stage” educational outcomes, where time spent in school and time spent on homework increased, but time spent in low-quality private tuition decreased. These results are likely driven by improvement in student motivation and changes in education-related behavior, as students who were now potentially substituting costly private tuition with improved time allocation for self-study and school-based study with the help of laptops. Furthermore, we also find that students exposed to the laptop program were now likely to have better comprehension and understanding of language, which potentially helped them understand math problems in a better manner, thereby resulting in improved foundational proficiency in mathematics (Abedi and Lord, 2001; Beal et al., 2010; Brown et al., 2011; Henry et al., 2014; Kieffer et al., 2009; Martiniello, 2008; Staley, 2005).

Third, our study highlights the empowering role of technology in closing socioeconomic as well as gender gaps in education, which is in contrast to some of the existing work (Hall et al., 2021). We show that students from resource-constrained backgrounds received much larger benefits from the program when compared to students from economically well-off households. Similarly, we find suggestive evidence that the “reverse” gender gap (in favor of girls) in foundational math skills closed on account of access to laptops, as boys experienced higher gains and caught up.

To the best of our knowledge, this is the first study that examines the impact of a government laptop program in India. Compared to free laptop programs implemented in other countries, the scale of the program evaluated here was much larger. For instance, the OLPC program (as discussed before) distributed over 3 million laptops in over 64 countries between 2005 and 2021. In contrast, the Tamil Nadu Free Laptop initiative provided laptops to over 2 million students in the first 3 years of the program in a single state of India.^4^^,^^5^

## Background

### Hypotheses

We hypothesize that since technological resources such as laptops form an important input for the education production function, access to them can have potential impacts on educational outputs such as the mathematical proficiency of students (Hanushek, 1979). Laptops provide students with access to a wide range of educational resources, with the flexibility of being able to access them when, where, and at the pace at which they would like. Particularly in the context of LMICs, where school classroom contexts can have a large number of students per teacher, more private learning afforded by laptops has the potential to improve learning outcomes. However, a lack of digital skills or other reasons that limit the ability to leverage the educational resources offered by laptops may limit the realization of these impacts, and in turn, result in null effects. Conversely, the direction of the impacts may also be negative if students who gain access to laptops use them for other purposes, such as entertainment or leisure, which may, in turn, reduce time spent on education. In sum, the direction of the impact of laptops can be positive or negative depending on how students use these resources.

### Tamil Nadu Free Laptop Scheme

The “Free Laptop Scheme” or “TFLS” was introduced in the southern state of Tamil Nadu in India in the year 2011 (marked in blue in Appendix Fig. A.1). It was the first scheme of its kind in the country that sought to provide free laptops to students studying in government or government-aided higher secondary schools and colleges.^6^ The stated rationale behind the program was to invest in the human resource potential of the state and develop digital skills that could open new opportunities to participate in the information technology labor market for youth. With the government providing free laptops only to students in government schools, it also aimed to bridge the digital divide between students studying in government and private schools, as the former are likely to be resource-constrained and underinvest in educational resources.^7^ Interestingly, the scheme was initially announced as a poll promise in the run-up to the 2011 Tamil Nadu elections by the All India Anna Dravida Munnetra Kazhagam Party, in which they eventually formed the government.^8^ The incumbent ruling party, Dravida Munnetra Kazhagam, also had a similar policy in their manifesto for the elections.^9^

The laptops provided to students under this scheme were procured by the state government through the Electronics Corporation of Tamil Nadu which issued a tender for the same. The laptops included a dual-boot operating system (Windows and Linux), as well as anti-virus software. It also included pre-installed educational content from the education department (Special Programme Implementation Department, 2019).^10^ The laptops were also marked with the state government logo for clear identification and traceability.^11^ Laptops provided under the program also came with a user manual as well as an instruction set in both English and Tamil languages. The head of a recipient institution was given the administrative responsibility to compile and maintain the list of students studying in grades that were covered by the program in a particular year.

The distribution of laptops under the program began simultaneously across all districts of Tamil Nadu in September 2011 (Appendix Figs. A.2 and A.3). In the initial phase of the program (2011–12 to 2013–14), higher secondary students studying in class 12th (final year of senior secondary school) as well as students studying in undergraduate degree programs (in different years) in government institutions were eligible for free laptops. The policy was implemented annually; however, significant changes occurred in policy guidelines with even class 11th becoming eligible for laptops starting the year 2018–19.^12^ Estimates suggest that the state government incurred an expenditure of over USD 600 million in the first 3 years of the program during which period they distributed over 2 million laptops.^13^ However, some media reports on the status of the program suggested that some laptops were sold off by the beneficiaries in the grey markets.^14^ Despite this, some media reports also mentioned that students perceived the scheme to have a positive impact on their academic and technical skills.^15^

## Empirical framework

### Data

To understand the potential impact of access to laptops on the math skills of students, we make use of the ASER survey conducted by Pratham, an Indian non-governmental organization established in 1995. The ASER survey is a state as well as nationally representative household survey that provides information on learning outcomes of around 600,000 school-aged rural children annually.^16^^,^^17^ We use data from the surveys conducted in 2008, 2009, 2010, 2011, 2012, 2013, and 2014 among all the states in the country for our analysis (ASER, 2008, 2009, 2010, 2011, 2012, 2013, 2014).^18^ The ASER Survey has previously been used in numerous studies in the education literature to study learning and educational outcomes in India (Adukia, 2017; Chakraborty and Jayaraman, 2019; Das and Sarkhel, 2023; Lahoti and Sahoo, 2020; Shah and Steinberg, 2019). It is important to note that the ASER survey by design provides only a “floor test” for the math ability of children as the questions asked test only the foundational skills in arithmetic.^19^A sample test in math is provided in Appendix Fig. A.4.

In addition to collecting information on foundational math skills, the survey also collects information on several individual, household as well as village-level characteristics. For instance, the survey includes information on the grade/class as well as the type of school (government or private) that a student is studying in, which is also useful for the purpose of our identification strategy. While the nature of the data constrains us to study impacts only for rural children, it is also helpful in the sense that inaccessibility to technological resources such as laptops is likely to be higher in this setting.

Table 1 provides the summary statistics for our primary outcome as well as the control variables. As can be observed in Table 1, the mean math score for the sample is 3.686, which seems to be on the higher side. However, this has to be seen in the context that students from classes 11th and 12th are being tested at much below their grade level in basic math and foundational arithmetic skills. Even for this below-grade-level outcome, we find that around 23% of students in these classes are not at the highest proficiency level, i.e., the ability to do division. The number becomes even higher when we look at students from the most economically deprived sections (proxied by housing quality) (27%).^20^

**Table 1:** Summary statistics.

			Pre-policy		Post-policy	
	Description	All	Ineligible	Eligible	Ineligible	Eligible
		Sample	Cohort	Cohort	Cohort	Cohort
*Math Score*	Can’t do any math = 0	3.686	3.796	3.815	3.555	3.655
	Recognize Numbers (1–9) = 1,	(0.621)	(0.512)	(0.501)	(0.707)	(0.639)
	Recognize Numbers (11–99) = 2, Subtraction = 3, Division = 4)					
*Child*’*s Age*	Continuous	15.731	15.702	15.834	15.694	15.842
	Variable	(0.805)	(0.847)	(0.831)	(0.745)	(0.835)
*Child’s Sex*	Male = 0	0.553	0.534	0.563	0.550	0.603
	Female = 1	(0.497)	(0.498)	(0.496)	(0.497)	(0.489)
*Mothers’s Age*	Continuous	37.253	36.993	37.430	37.224	37.917
	Variable	(5.333)	(5.747)	(5.771)	(4.894)	(5.017)
*Mother Attended School*	No = 0	0.629	0.614	0.609	0.643	0.645
	Yes = 1	(0.482)	(0.486)	(0.488)	(0.478)	(0.478)
*Total Members in household*	Continuous	4.825	4.849	4.895	4.802	4.767
	Variable	(1.535)	(1.340)	(2.058)	(1.308)	(2.055)
*House Type*	Pucca House = 1	0.596	0.375	0.370	0.792	0.794
	No Pucca House = 0	(0.490)	(0.484)	(0.483)	(0.405)	(0.404)
*Household has Electricity Connection*	No = 0	0.976	0.965	0.971	0.983	0.988
	Yes = 1	(0.151)	(0.181)	(0.165)	(0.128)	(0.108)
*Use of Electricity observed in hh on Interview Day*	No = 0	0.898	0.908	0.923	0.880	0.902
	Yes = 1	(0.302)	(0.288)	(0.265)	(0.324)	(0.296)
*Electricity in Village*	No = 0	0.991	0.993	0.992	0.990	0.989
	Yes = 1	(0.092)	(0.083)	(0.084)	(0.097)	(0.102)
*Bank in Village*	No = 0	0.401	0.398	0.455	0.393	0.382
	Yes = 1	(0.490)	(0.489)	(0.498)	(0.488)	(0.486)
*Primary Govt. School in Village*	No = 0	0.869	0.782	0.784	0.935	0.940
	Yes = 1	(0.337)	(0.412)	(0.411)	(0.245)	(0.236)
*Middle Govt*. *School in Village*	No = 0	0.489	0.539	0.495	0.461	0.452
	Yes = 1	(0.499)	(0.498)	(0.500)	(0.498)	(0.498)
*Secondary Govt*. *School in Village*	No = 0	0.242	0.338	0.349	0.166	0.166
	Yes = 1	(0.428)	(0.473)	(0.477)	(0.372)	(0.372)
*Private School in Village*	No = 0	0.285	0.319	0.318	0.261	0.250
	Yes = 1	(0.451)	(0.466)	(0.466)	(0.439)	(0.433)

The table shows summary statistics, mean and standard deviation (in parentheses), using the ASER dataset.

We also supplement our analysis with the data from the India Human Development Survey (IHDS) Round 1 and 2 conducted during 2004–05 and between 2011 and 2013, respectively, to tease out potential mechanisms for our effects (Desai and Vanneman, 2018; Desai et al., 2018, 2019). The survey is nationally representative in nature and covers over 40,000 households in each wave. It is conducted jointly by the University of Maryland and the National Council of Applied Economic Research. The survey collects information on education-related outcomes of children and has also been used extensively in the education and development studies literature (Chatterjee and Poddar, 2021; Kaur, 2021; Singh and Shemyakina, 2016).

### Identification strategy

In trying to understand the potential impact of access to laptops on the foundational math skills of students, we are essentially looking at the following functional relationship:

$$Y=f(Laptop,{X}_{obs},{X}_{unobs})$$

This implies that the math skills of students (*Y*) are a function of laptop access coupled with several observable and unobservable factors. This makes sense as technological resources such as computers and laptops can potentially constitute important inputs to the education production function that can have impacts on the educational outputs, such as learning and performance of students (Hanushek, 1979). However, estimation of the above-mentioned functional relationship using ordinary least squares is challenging on account of endogeneity concerns. For instance, students or families having access to laptops might be characteristically different from those not having this access. Similarly, while laptops can affect learning outcomes, the decision to purchase a laptop can also be affected by or be conditional on learning outcomes.

In order to address endogeneity concerns, we use a quasi-experimental design to understand the potential impact of laptops on the academic proficiency of students. More specifically, we use plausibly exogenous variation generated by the institutional features of the “TFLS” in India to examine the potential impacts of technology on students’ foundational math skills. Under the program, free laptops were provided by the Tamil Nadu State government to students studying in class 12th of the public (or government) schools in the state starting in the year 2011. We exploit these institutional features of the policy to set up a double difference design (difference-in-difference or DD) in order to understand the causal impact of the program on the foundational math skills of students in Tamil Nadu. The two dimensions of our double difference framework come from *cohort* and *time* variation. Under our framework, students in class 11th form the *ineligible* *cohort* as they did not receive free laptops, whereas students in class 12th form the *eligible* *cohort* as they received the program benefits. Similarly, since the policy was introduced in the year 2011, time periods before it are considered as *pre*-*period,* and after it (including 2011) are considered as *post-period*.

In using our double difference design, we are essentially comparing the foundational math proficiency of our *eligible cohort* (class 12th) to that of the *ineligible cohort* (class 11th), in the state of Tamil Nadu, *before* and *after* the program. More specifically, we study the effect of being exposed to the TFLS using an intent-to-treat analysis by running the regression equation given below for each student *i* belonging to household *h* from village *v* in Tamil Nadu. We restrict our analysis to students studying in government schools, as the scheme was only available to them.

1 $$\begin{array}{l}{Y}_{ihv}={\delta }_{t}+{\beta }_{1}\cdot (Eligible\times Post)+{\beta }_{2}\cdot (Eligible)\\\qquad\,+\,{\beta }_{3}\cdot (Post)+{\gamma }_{1}\cdot {X}_{i}+{\gamma }_{2}\cdot {X}_{h}+{\gamma }_{3}\cdot {X}_{v}+{\epsilon }_{ihv}\end{array}$$

In Equation (1), *δ*_*t*_ captures time fixed effects, and *Y*_*ihv*_ captures the foundational math proficiency of students. *Eligible* is a dummy variable that takes the value 1 for the eligible cohort (Class 12th) who received the program benefits and 0 for the ineligible cohort, i.e., class 11th. The dummy variable *Post* takes the value 1 for the period post-policy introduction and zero in the pre-policy period. *β*_1_, then, is our coefficient of interest and picks out the effect of the TFLS program on foundational math proficiency of students in Tamil Nadu. We also use several individual, household, and village-level controls for our analysis. These include age, gender, mother’s schooling status, mother’s age, number of household members, electricity connection in the household, electricity in the household on the day of interview (observed use), type of household, electricity in the village, bank in the village, and availability of a primary, a middle, a secondary, and a private school in the village. We also use robust standard errors for our analysis.

**Fig. 1:**
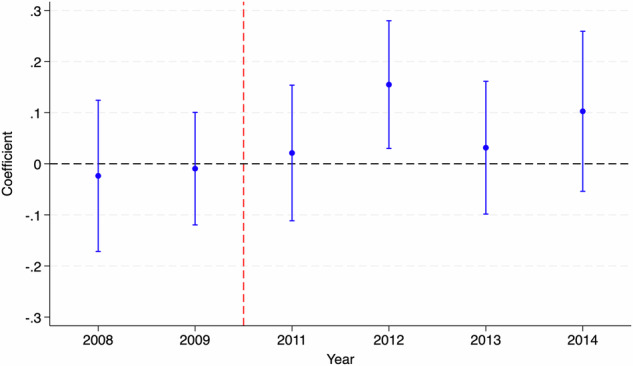
Dynamic double difference estimates (event study). The figure shows dynamic differences in difference estimates (along with 95% confidence interval) for math proficiency using ASER data. The year 2010 has been considered as the base year for the plot.

We test the identification assumption behind our double difference design using a parallel trends test for the pre-policy period (2008–2010) (Muralidharan and Prakash, 2017). The results for the same are reported in Table 2. We can observe that the null hypothesis of parallel trends cannot be rejected across a variety of specifications. The effect sizes are close to zero as well as insignificant at conventional levels. We also plot an event study graph (or dynamic difference-in-difference) in Fig. 1 with the year 2010 as the base. As can be observed, we do not find evidence of any existing pre-trends.

**Table 2:** Impact on math proficiency: testing for parallel trends (DD).

	(1)	(2)	(3)	(4)	(5)
*Eligible* × *Year*	−0.011	0.011	−0.001	0.016	0.015
	(0.025)	(0.025)	(0.028)	(0.017)	(0.035)
*Eligible*	22.722	23.374	3.908	−33.083	−30.894
	(51.187)	(52.000)	(56.926)	(70.849)	(71.074)
*Year*	0.019	0.016	0.007	0.022	0.021
	(0.012)	(0.012)	(0.013)	(0.017)	(0.017)
*R*^2^	0.001	0.02	0.03	0.06	0.06
Observations	3268	3164	2784	1986	1986
Individual Controls	No	Yes	Yes	Yes	Yes
Household Controls	No	No	Yes	Yes	Yes
Village Controls	No	No	No	Yes	Yes
Year Fixed Effects	No	No	No	No	Yes

All columns represent different regressions for testing the parallel trends assumption in policy pre-period (2008–2010) using the ASER dataset. Control variables included as part of different specifications include age, gender, mother’s schooling status, mother’s age, number of household members, electricity connection in the household, electricity in the household on the day of interview (observed use), type of household, electricity in the village, bank in the village, and availability of a primary school, a middle school, a secondary school, and a private school in the village. Robust standard errors are reported in parentheses.

*p < 0.1, **p < 0.05, ***p < 0.01.

## Results

In this section, we first report the results for the potential impact of access to laptops through TFLS on the foundational math skills of students. This is then followed by an analysis of heterogeneous impacts of the policy across the dimensions of wealth status and gender. We then perform a variety of robustness checks to ensure that our double difference identification design is picking up the effect of the policy and not producing a spurious result.

### Main findings

We report the results from our double difference identification design in Table 3. The results suggest that the potential exposure to the TFLS program had a positive effect on the foundational math proficiency of students. We, specifically, observe that the math scores are around 0.085 points higher for students who were potentially exposed to the program. This translates to an effect size of 2.3% when compared to the mean. The result implies that access to laptops can potentially help in improving basic foundational math skills of higher secondary students. We also observe in the dynamic double difference graph in Fig. 1 that our results are driven by improvement in foundational math proficiency of students in the years 2012 and 2014. This makes sense as the implementation of the TFLS program only began in September 2011. We also observe negligible effects for the year 2013, which might potentially be on account of the differential distribution cycle for the laptop program in different years.

**Table 3:** Impact on math proficiency: DD estimates.

	(1)	(2)	(3)	(4)	(5)
*Eligible* × *Post*	0.081**	0.085***	0.094***	0.076**	0.084**
	(0.033)	(0.033)	(0.034)	(0.038)	(0.038)
*Eligible*	0.019	0.007	−0.002	0.002	−0.001
	(0.020)	(0.021)	(0.034)	(0.026)	(0.025)
*Post*	−0.240***	−0.239***	−0.270***	−0.271***	−0.491***
	(0.017)	(0.017)	(0.018)	(0.021)	(0.040)
*R*^2^	0.03	0.04	0.05	0.05	0.08
Observations	6743	6626	6053	4920	4920
Individual Controls	No	Yes	Yes	Yes	Yes
Household Controls	No	No	Yes	Yes	Yes
Village Controls	No	No	No	Yes	Yes
Year Fixed Effects	No	No	No	No	Yes

All columns represent different regressions for the impact of the TFLS program on the foundational math skills of students using the ASER dataset. Control variables included as part of different specifications include age, gender, mother’s schooling status, mother’s age, number of household members, electricity connection in the household, electricity in the household on the day of interview (observed use), type of household, electricity in the village, bank in the village, and availability of a primary school, a middle school, a secondary school, and a private school in the village. Robust standard errors are reported in parentheses.

*p < 0.1, **p < 0.05, ***p < 0.01.

We further assess the levels at which these learning improvements happen for mathematics when students get access to laptops. For this purpose, we estimate a linear probability model where our dependent variable now becomes a dummy which takes the value 1 if the student has achieved skill *atleast* at a certain level of learning in mathematics (Chakraborty and Jayaraman, 2019; Chatterjee and Poddar, 2021; Lahoti and Sahoo, 2020). This alternative formulation also has the advantage of addressing the issue of ordinality of math proficiency scores, which we use for our primary analysis. This is because the variable capturing proficiency scores is continuous in nature and can, therefore, be affected by the choice of scale. Using a linear probability model helps us to account for this concern as well.

Table 4 reports the results for this exercise to understand the levels of learning. We observe that the impact of access to laptops is much higher for higher levels of learning, such as subtraction and division, when compared to lower levels of learning. Students who were potentially exposed to the program were 5% (when compared to the mean) more likely to be proficient in subtraction when compared to students who were not exposed to the program. While the coefficient for division is imprecisely estimated, we still find that exposed students were 4.6% more likely to be proficient in it when compared to students not exposed to the policy. We do not find improvement in lower levels of learning, as the coefficients are much closer to zero.

**Table 4:** Impact on math proficiency: levels of learning (DD).

Proficiency level	Numbers (1–9)+	Numbers (11–99)+	Subtraction+	Division+
Proficiency score	≥1	≥2	≥3	=4
	(1)	(2)	(3)	(4)
*Eligible* × *Post*	−0.002	0.005	0.046***	0.035
	(0.003)	(0.004)	(0.016)	(0.026)
*Eligible*	−0.000002	−0.004	−0.013	0.015
	(0.002)	(0.003)	(0.009)	(0.018)
*Post*	−0.0003	0.0006	−0.183***	−0.308***
	(0.001)	(0.004)	(0.018)	(0.027)
*R*^2^	0.02	0.03	0.06	0.08
Observations	4920	4920	4920	4920
Controls	Yes	Yes	Yes	Yes
Year Fixed Effects	Yes	Yes	Yes	Yes

All columns represent different regressions for the impact of the TFLS program on the foundational math skills of students using the ASER dataset. Control variables included as part of different specifications include age, gender, mother’s schooling status, mother’s age, number of household members, electricity connection in the household, electricity in the household on the day of interview (observed use), type of household, electricity in the village, bank in the village, and availability of a primary school, a middle school, a secondary school, and a private school in the village. Robust standard errors are reported in parentheses.

*p < 0.1, **p < 0.05, ***p < 0.01.

### Sub-sample analysis and heterogeneity

The education literature from India suggests that educational inequalities persist in the country in terms of gender, caste, religion, and economic status (Asadullah and Yalonetzky, 2012; Borooah, 2012; Desai and Kulkarni, 2008; Gandhi Kingdon, 2002; Kingdon, 2007; Varughese and Bairagya, 2020). Hence, it is possible that the heterogeneous effects of the Tamil Nadu Free Laptop program on math skills may exist based on beneficiary students’ individual attributes as well as social and religious affiliation. Unfortunately, our data does not provide us with information on the caste and religion of the student. In the next subsections, we therefore focus on the economic status as well as the gender of the student to understand potential impacts of the policy based on these attributes.

#### Economic status

Our reduced form analysis suggests that access to technological resources such as laptops can help to improve the math skills of students. As these resources are less affordable for students coming from resource-constrained households, the provision of free laptops to students from these backgrounds can have much larger impacts when compared to students coming from economically better-off households that might already have access to these resources. However, it is also possible that poor households receiving laptops consider it as a one-off economic opportunity to benefit from the sale of the asset. In this case, laptops will have much smaller, if any, impacts on students from these backgrounds.

**Fig. 2:**
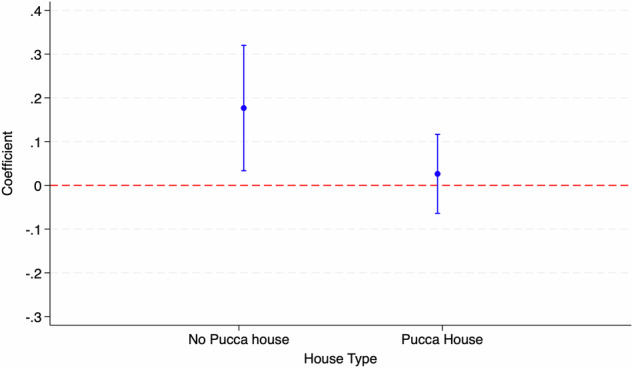
Sub-sample analysis for housing quality. The figure shows sub-sample estimates (along with 95% confidence interval) for math proficiency for students residing in a “Pucca house” or “No Pucca house” using ASER data.

The ASER data does not provide us with direct information on the economic status of the student. However, existing literature on India suggests that information on housing assets serves as a good proxy for the same (Chakraborty and Jayaraman, 2019). We, therefore, use data on housing quality to understand heterogeneity by the economic status of the student. Here, we focus on whether a student stays in a “Pucca house” or not, where “Pucca house” refers to durable houses made of burnt bricks and cement. We conjecture that a student living in a “Pucca house” is likely to be economically well off in a relative sense when compared to a student living in a “Kutcha or Semi-Pucca” house. Figure 2 plots the coefficients for sub-sample analysis for two different house types. We find that students not living in “Pucca house” and belonging to economically poorer households were the primary beneficiaries of the program in terms of their learning outcomes. Students living in “Pucca houses,” who were also likely to be much better off economically, experienced no positive effects of the TFLS program.

#### Gender

We then examine if access to laptops on account of the TFLS had any differential impacts on the math scores of boys and girls. Existing literature points towards differential use of computers for boys and girls that can potentially result in differential effects (Fairlie, 2016; Fairlie and Robinson, 2013). Also, in the Indian setting, where girls continue to experience educational disadvantage in school classroom settings, more focused and personalized access to resources through laptops has the potential to provide larger payoffs (Adukia, 2017; Chatterjee and Poddar, 2021; Gandhi Kingdon, 2002; Kingdon, 2007). Furthermore, if girls start from a lower base in math skills when compared to boys, the marginal impacts of access to laptops can be much higher for them. On the contrary, if boys start from a lower base, the marginal impacts for them are likely to be higher when compared to girls (Contini et al., 2017; Fryer and Levitt, 2010; Welmond and Gregory, 2021). The literature also suggests that in a resource-constrained setting, women and girls might be less likely to retain control over technological assets such as mobiles and laptops, as the control might pass on to a male family member or the asset might be sold off (Roessler et al., 2021). If that happens, improved access to laptops through TFLS will result in much lower average positive impact on the math skills of girls when compared to boys.

We, therefore, perform a sub-sample analysis for the potential impact of the program on the math skills of boys and girls in Table 5. As we can observe from the sub-sample analysis in Column (1) and Column (2), the math skills of both boys and girls seemingly improved on account of the TFLS. However, the coefficient for boys is almost double when compared to that of the girls. When we investigate further, we find that this difference in coefficient is indeed driven by economically constrained households not living in “pucca houses” (Table 6). Hence, it is possible that differential selling-off of laptops provided to girls by economically constrained households can potentially be one of the reasons for lower effects experienced by them. This also makes sense considering that anecdotal evidence on the policy from media reports suggests that several laptops distributed under the scheme do appear on the grey market; however, we do not have enough information on whether this was more prominent for girl beneficiaries.^21^^,^^22^

**Table 5:** Sub-sample analysis: boys vs girls.

	Boys	Girls
	(1)	(2)
*Eligible* × *Post*	0.112*	0.058
	(0.064)	(0.047)
*Eligible*	−0.042	0.028
	(0.042)	(0.032)
*Post*	−0.564***	−0.445***
	(0.068)	(0.051)
*R*^2^	0.10	0.08
Observations	2145	2775
Controls	Yes	Yes
Year Fixed Effects	Yes	Yes

The table shows results from regressions using the ASER dataset. Control variables included as part of the regression are age, gender, mother’s schooling status, mother’s age, number of household members, electricity connection in the household, electricity in the household on the day of interview (observed use), type of household, electricity in the village, bank in the village, and availability of a primary school, a middle school, a secondary school, and a private school in the village. Robust standard errors are reported in parentheses.

*p < 0.1, **p < 0.05, ***p < 0.01.

**Table 6:** Sub-sample analysis: boys vs girls across wealth status.

	No Pucca house		Pucca house	
	Boys	Girls	Boys	Girls
	(1)	(2)	(3)	(4)
*Eligible* × *Post*	0.234*	0.159*	0.022	0.024
	(0.125)	(0.090)	(0.074)	(0.058)
*Eligible*	−0.099	0.003	0.033	0.049
	(0.061)	(0.046)	(0.049)	(0.043)
*Post*	−0.740***	−0.606***	−0.455***	−0.380***
	(0.123)	(0.106)	(0.096)	(0.065)
*R*^2^	0.15	0.12	0.09	0.07
Observations	807	992	1,338	1,783
Controls	Yes	Yes	Yes	Yes
Year Fixed Effects	Yes	Yes	Yes	Yes

The table shows results for the impact of TFLS on boys and girls based on wealth status using the ASER dataset. Control variables included as part of the regression are age, gender, mother’s schooling status, mother’s age, number of household members, electricity connection in the household, electricity in the household on the day of interview (observed use), type of household, electricity in the village, bank in the village, and availability of a primary school, a middle school, a secondary school, and a private school in the village. Robust standard errors are reported in parentheses.

*p < 0.1, **p < 0.05, ***p < 0.01.

Another potential factor that could have led to lower marginal positive effects on math scores for girls when compared to boys may be their higher starting base level in the subject. While evidence at the global level suggests that girls lag behind boys in math outcomes, evidence contrary to the same is also present for some countries and contexts.^23^ (Contini et al., 2017; Fryer and Levitt, 2010; Welmond and Gregory, 2021). In the case of India, evidence indicates a gender gap in mathematics for predominantly North Indian States and, in contrast, a “reverse” gender gap where boys lag behind girls in predominantly South Indian states, including Tamil Nadu (Das and Singhal, 2023). Das and Singhal (2023) also rely on the ASER dataset for their analysis, as is the case in this study. When we look at our data, we find that boys (pre-policy mean math score = 3.790) studying in class 12th of government schools in Tamil Nadu lag behind in mathematics when compared to girls (pre-policy mean math score = 3.835) in the same cohort. Hence, higher positive impacts of the Tamil Nadu Free Laptop program for boys can also potentially reflect boys catching up with girls in terms of math scores, as they start from a lower base.

### Robustness checks

#### Cohort falsification

Our double difference identification strategy relies on the use of cohort variation to pick out the effects of the TFLS program. Essentially, we are comparing the foundational math proficiency of students in class 12th (eligible cohort) with those in class 11th (ineligible cohort) and saying that in the absence of the program, the difference between the outcomes of these two cohorts would be zero. To test the validity of this identification assumption, we conduct a falsification check by comparing the outcomes of class 11th with those of class 10th students using the regression specification provided in Equation (1). Here, we provide a *fake* *treatment* to class 11th students and compare their outcomes to class 10th students. To do this, we generate a dummy variable “*Class 11*” which takes the value 1 if the student is in Class 11 of the government school and zero if student is in Class 10 of the government school. Since both these cohorts were essentially ineligible for the program, ideally, we should not find their foundational math skills to be statistically different from each other.

We report the results for this controlled experiment in Table 7 (Duflo, 2001). As expected, we do not find any statistical difference between the math learning outcomes of class 11th and class 10th students in Tamil Nadu. This gives us confidence that our double difference identification design is picking up the true causal effect of the TFLS program.

**Table 7:** Impact on math proficiency: cohort falsification (DD).

	Math score
	(1)
*Class 11* × *Post*	0.004
	(0.026)
*Class 11*	0.128
	(0.020)
*Post*	−0.499
	(0.032)
*R*^2^	0.07
Observations	10,294
Controls	Yes
Year Fixed Effects	Yes

The table shows results from regressions using the ASER dataset. Control variables included as part of the regression are age, gender, mother’s schooling status, mother’s age, number of household members, electricity connection in the household, electricity in the household on the day of interview (observed use), type of household, electricity in the village, bank in the village, and availability of a primary school, a middle school, a secondary school, and a private school in the village. Robust standard errors are reported in parentheses.

*p < 0.1, **p < 0.05, ***p < 0.01.

#### Alternative control group

We also try to test the sensitivity of our estimates to alternative definitions of the control group, i.e., the ineligible cohort. To do this, we now pool class 10 students and class 11 students together as a combined control group, i.e., the ineligible cohort, and compare their outcomes with the eligible treated group, i.e., class 12 students, before and after the program implementation in Tamil Nadu. The results for this exercise are reported in Table 8. As we can observe, the coefficients from the exercise are very similar to our main estimates, thereby providing us confidence that our identification strategy is correctly picking up the effect of the program.

**Table 8:** Impact on math proficiency: DD estimates after including class 10th as control group.

	(1)	(2)	(3)	(4)	(5)	(6)
*Eligible* × *Post*	0.065**	0.068**	0.077**	0.069*	0.085**	0.087**
	(0.030)	(0.031)	(0.032)	(0.036)	(0.036)	(0.035)
*Eligible*	0.120***	0.072***	0.060***	0.062**	0.059**	0.121***
	(0.018)	(0.019)	(0.021)	(0.025)	(0.025)	(0.028)
*Post*	−0.225***	−0.223***	−0.265***	−0.269***	−0.481***	−0.491***
	(0.011)	(0.011)	(0.012)	(0.015)	(0.028)	(0.028)
*R*^2^	0.03	0.04	0.05	0.05	0.07	0.07
Observations	15,832	15,604	14,209	11,427	11,427	11,427
Individual Controls	No	Yes	Yes	Yes	Yes	Yes
Household Controls	No	No	Yes	Yes	Yes	Yes
Village Controls	No	No	No	Yes	Yes	Yes
Year Fixed Effects	No	No	No	No	Yes	Yes
Cohort Fixed Effects	No	No	No	No	No	Yes

The table shows results from regressions using the ASER dataset. Control variables included as part of different specifications include age, gender, mother’s schooling status, mother’s age, number of household members, electricity connection in the household, electricity in the household on the day of interview (observed use), type of household, electricity in the village, bank in the village, and availability of a primary school, a middle school, a secondary school, and a private school in the village. Robust standard errors are reported in parentheses.

*p < 0.1, **p < 0.05, ***p < 0.01.

#### Selection into government schools

A potential concern for our analysis is if the program altered the enrollment in government vis-a-vis private schools. This is possible as students might switch from private to government schools to gain access to program benefits. This has the potential to introduce selection bias in our results, as inclusion in our sample is conditional on a student studying in a government school. We, therefore, test if the introduction of the program impacted the likelihood of individuals enrolling in government vis-a-vis private schools and present our results in Table 9. The results suggest that the program did not have any impact on the likelihood of students enrolling in a government school when compared with a private school.

**Table 9:** Impact on the likelihood of enrolling in government school (DD).

	Government school
	(1)
*Eligible* × *Post*	0.019
	(0.024)
*Eligible*	−0.023
	(0.018)
*Post*	−0.004
	(0.021)
*R*^2^	0.05
Observations	6841
Controls	Yes
Year Fixed Effects	Yes

The table shows results for the impact of TFLS on selection into government schools using the ASER dataset. Control variables included as part of the regression are age, gender, mother’s schooling status, mother’s age, number of household members, electricity connection in the household, electricity in the household on the day of interview (observed use), type of household, electricity in the village, bank in the village, and availability of a primary school, a middle school, a secondary school, and a private school in the village. Robust standard errors are reported in parentheses.

*p < 0.1, **p < 0.05, ***p < 0.01.

#### Alternative strategy: triple difference

In this section, we devise an alternative triple difference strategy to test the robustness of our results estimated using a double difference design (Gruber, 1994). Here, we introduce a third dimension to our existing double difference framework in the form of a *state* variation. This means that we now compare the foundational math proficiency of our *eligible cohort* (class 12th) to that of the *ineligible cohort* (class 11th) across our *treated state* of Tamil Nadu and other *control states* in India, *before* and *after* the program.^24^^,^^25^ Other than informing about the sensitivity of our main estimates, an additional advantage of using this alternative strategy is that it enables us to explore potential channels, using both ASER and IHDS datasets, through which the TFLS may impact foundational math skills. This is because it increases the statistical power and the precision with which we can pick the effects on account of a larger sample size accorded by the strategy. For instance, the data on English language competency that we explore as a potential channel is only available in the years 2009, 2012, and 2014. Similarly, the state-level sample in IHDS is quite small from the perspective of performing intra-state cohort-level analysis using a difference-in-difference design. The usage of the triple difference design here helps us to increase the precision with which we can pick out the effects of the TFLS on these intermediate variables that serve as potential channels for our effects.

In order to perform a triple difference analysis, we run the regression equation given below for each student *i* belonging to household *h* from village *v* in state *s*. As before, we restrict our analysis to students studying in government schools, as the scheme was only available to them.

2 $$\begin{array}{l}{Y}_{ihvs}={\alpha }_{s}+{\delta }_{t}+\,{\theta }_{1}\cdot (Eligible\times Treated\times Post)+{\theta }_{2}\cdot (Eligible\times Treated)\\\qquad\qquad\;\;+\,{\theta }_{3}\cdot (Eligible\times Post)+{\theta }_{4}\cdot (Treated\times Post)+{\theta }_{5}\cdot (Eligible)\\\qquad\qquad\;\;+\,{\theta }_{6}\cdot (Treated)+{\theta }_{7}\cdot (Post)+{\rho }_{1}\cdot {X}_{i}+{\rho }_{2}\cdot {X}_{h}+{\rho }_{3}\cdot {X}_{v}+{\epsilon }_{ihvs}\end{array}$$

In Equation (2), *α*_*s*_ and *δ*_*t*_ capture state fixed effects and time fixed effects, respectively. *Treated* is a dummy variable that takes the value 1 for the treated state of Tamil Nadu and zero, otherwise. *Eligible* and *Post* refer to dummies for the eligible cohort and post-policy period, respectively. Here, *θ*_1_ is our coefficient of interest that picks out the effect of the TFLS program on the foundational math proficiency of students. We also use robust standard errors clustered at the state level for our analysis here.

**Fig. 3:**
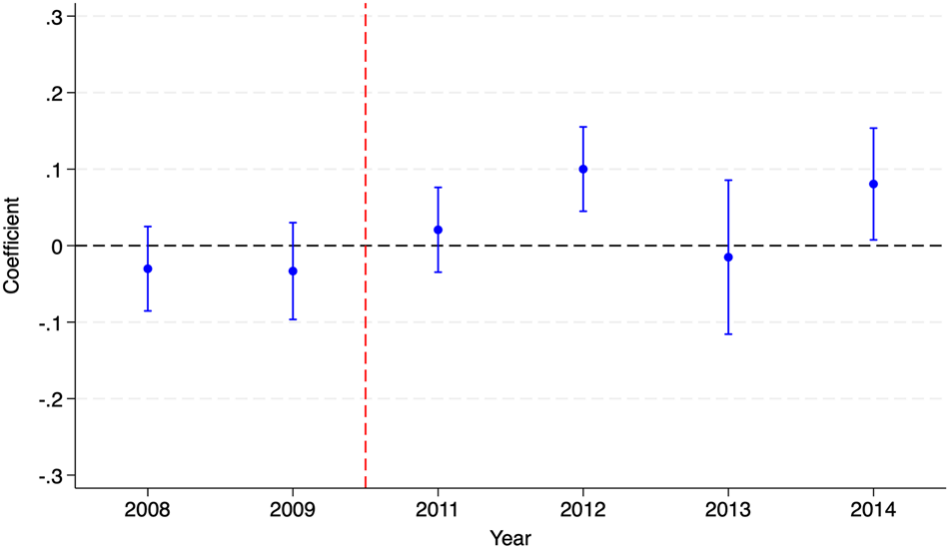
Dynamic triple difference estimates (event study). The figure shows dynamic triple difference estimates (along with 95% confidence interval) for Math Proficiency using ASER data. The year 2010 has been considered as the base year for the plot.

Using Table 10 and Fig. 3, we first show that the parallel trends assumption for the triple difference model is satisfied (Muralidharan and Prakash, 2017). We then present the results from our triple difference design in Table 11. As can be observed, the results look very similar to our primary estimates using a double difference design. This gives us confidence that our primary strategy, based on a double difference design, is correctly picking the impacts of TFLS. As stated before, we use this triple difference design to study potential channels for our effects on foundational math proficiency in the next section.

**Table 10:** Impact on math proficiency: parallel trends for DDD estimates.

	(1)	(2)	(3)	(4)	(5)	(6)
*Eligible* × *Treated* × *Year*	−0.012	−0.012	−0.005	0.008	0.012	0.012
	(0.015)	(0.015)	(0.010)	(0.013)	(0.012)	(0.012)
*Eligible* × *Treated*	24.722	25.519	11.964	−16.915	−24.786	−25.323
	(29.141)	(31.264)	(21.965)	(26.494)	(26.038)	(24.901)
*Treated* × *Year*	0.024**	0.019**	0.012	0.018*	0.023**	0.022**
	(0.009)	(0.008)	(0.010)	(0.010)	(0.009)	(0.009)
*Eligible* × *Year*	0.001	0.002	0.005	0.0048	0.0001	−0.003
	(0.014)	(0.015)	(0.011)	(0.013)	(0.012)	(0.011)
*Eligible*	−1.999	−5.689	−11.741	−9.627	−0.374	6.886
	(29.141)	(31.676)	(22.129)	(27.298)	(25.896)	(23.912)
*Year*	−0.005	−0.003	−0.005	0.001	−0.003	−0.004
	(0.009)	(0.008)	(0.009)	(0.009)	(0.009)	(0.008)
*Treated*	−49.111**	−38.364**	−24.799	−38.177*	–	–
	(19.621)	(16.559)	(21.572)	(21.889)	–	–
*R*^2^	0.001	0.10	0.11	0.11	0.13	0.11
Observations	18,047	17,317	15,318	12,633	12,633	30,255
Individual Controls	No	Yes	Yes	Yes	Yes	Yes
Household Controls	No	No	Yes	Yes	Yes	Yes
Village Controls	No	No	No	Yes	Yes	Yes
State Fixed Effects	No	No	No	No	Yes	Yes
Year Fixed Effects	No	No	No	No	No	Yes

All columns represent different regressions for testing the parallel trends assumption using the ASER dataset (2008–2010). Control variables included as part of different specifications include age, gender, mother’s schooling status, mother’s age, number of household members, electricity connection in the household, electricity in the household on the day of interview (observed use), type of household, electricity in the village, bank in the village, and availability of a primary school, a middle school, a secondary school, and a private school in the village. Robust standard errors clustered at the state level are reported in parentheses.

*p < 0.1, **p < 0.05, ***p < 0.01.

**Table 11:** Impact on math proficiency: DDD estimates.

	(1)	(2)	(3)	(4)	(5)	(6)
*Eligible* × *Treated* × *Post*	0.034	0.048*	0.055***	0.035*	0.061***	0.065***
	(0.024)	(0.025)	(0.018)	(0.018)	(0.021)	(0.019)
*Eligible* × *Treated*	−0.017	−0.023	−0.033**	−0.025*	−0.038**	−0.020
	(0.015)	(0.018)	(0.015)	(0.014)	(0.016)	(0.014)
*Treated* × *Post*	−0.040*	−0.033	−0.069***	−0.062***	−0.067***	−0.049**
	(0.021)	(0.021)	(0.020)	(0.020)	(0.021)	(0.022)
*Eligible* × *Post*	0.045*	0.037	0.041**	0.048**	0.027	0.023
	(0.024)	(0.025)	(0.019)	(0.018)	(0.020)	(0.018)
*Eligible*	0.036**	0.026	0.024*	0.020	0.034**	0.016
	(0.015)	(0.017)	(0.014)	(0.014)	(0.016)	(0.015)
*Post*	−0.200***	−0.203***	−0.199***	−0.198***	−0.318***	−0.354***
	(0.021)	(0.021)	(0.019)	(0.020)	(0.033)	(0.034)
*Treated*	−0.028	−0.056**	−0.049**	−0.039	−0.041*	–
	(0.025)	(0.024)	(0.023)	(0.023)	(0.024)	–
*R*^2^	0.02	0.07	0.07	0.08	0.09	0.11
Observations	41,952	41,037	35,148	30,255	30,255	30,255
Individual Controls	No	Yes	Yes	Yes	Yes	Yes
Household Controls	No	No	Yes	Yes	Yes	Yes
Village Controls	No	No	No	Yes	Yes	Yes
Year Fixed Effects	No	No	No	No	Yes	Yes
State Fixed Effects	No	No	No	No	No	Yes

All columns represent different regressions for the impact of the TFLS program on the foundational math skills of students using the ASER dataset. Control variables included as part of different specifications include age, gender, mother’s schooling status, mother’s age, number of household members, electricity connection in the household, electricity in the household on the day of interview (observed use), type of household, electricity in the village, bank in the village, and availability of a primary school, a middle school, a secondary school, and a private school in the village. Robust standard errors clustered at the state level are reported in parentheses.

*p < 0.1, **p < 0.05, ***p < 0.01.

#### Other concerns and checks

A potential concern with our analysis is that the ASER data only covers students up to the age of 16 years to collect information on foundational math proficiency. While students aged 16 years were eligible to be enrolled in class 12th and constituted a significant proportion of enrolled students in the grade both in 2012 and earlier years in Tamil Nadu (NUEPA, 2013a, 2013b), this creates a concern in that we are missing out on students from upper age cohorts, e.g., 17–18 years of age, who might have also been enrolled in class 12th and may be characteristically different from students aged 16 years.^26^ However, literature on the impact of age on learning outcomes suggests that this relationship is likely to be strong only in the early grades (Deming and Dynarski, 2008). Considering that we are looking at an outcome that is much below the grade level, the impact of the age of students studying in class 12th on our estimates is likely to be minimal. We further run a model, where we control for age fixed effects to account for time-invariant characteristics related to age. The results for this exercise are reported in Table 12 and are in line with our main estimates. We also conduct another check where we report estimates from a different specification where we cluster the standard errors at the district level and report the results in Table 13. As we can observe, the results are very similar to our main estimates and are robust to the use of bootstrap *p* values.

**Table 12:** Accounting for age fixed effects.

	(1)
*Eligible* × *Post*	0.086**
	(0.038)
*Eligible*	0.009
	(0.025)
*Post*	−0.498***
	(0.040)
*R*^2^	0.10
Observations	4920
Controls	Yes
Year Fixed Effects	Yes
Age Fixed Effects	Yes

The table shows results from regressions using the ASER dataset. Control variables included as part of the regression are age, gender, mother’s schooling status, mother’s age, number of household members, electricity connection in the household, electricity in the household on the day of interview (observed use), type of household, electricity in the village, bank in the village, and availability of a primary school, a middle school, a secondary school, and a private school in the village. Robust standard errors are reported in parentheses.

*p < 0.1, **p < 0.05, ***p < 0.01.

**Table 13:** Accounting for alternative specification.

	(1)
*Eligible* × *Post*	0.084*
	(0.045)
*Eligible*	−0.001
	(0.027)
*Post*	−0.491***
	(0.070)
*R*^2^	0.09
Bootstrap *P* Value	0.08
Observations	4920
Controls	Yes
Year Fixed Effects	Yes
Clustering Level	District

The table shows results from regressions using the ASER dataset. Control variables included as part of the regression are age, gender, mother’s schooling status, mother’s age, number of household members, electricity connection in the household, electricity in the household on the day of interview (observed use), type of household, electricity in the village, bank in the village, and availability of a primary school, a middle school, a secondary school, and a private school in the village. Robust standard errors clustered at the district level are reported in parentheses.

*p < 0.1, **p < 0.05, ***p < 0.01.

## Potential channels

The analysis in the preceding section provides us with a clear reduced form result that access to laptops has a positive impact on the foundational math skills of students. However, the underlying mechanisms behind this result still remain unclear. A good way to begin this enquiry into mechanisms would be to first test whether the TFLS actually resulted in improved ownership of laptops amongst students. This is important considering that the program is primarily aimed at improving laptop access among upper secondary students studying in government schools so that they are not left behind in the digital age. Unfortunately, we do not have information on laptop ownership in the ASER data. When it comes to the IHDS data, we have information on laptop ownership but only for the post-policy period (2011–2012). This does not allow us to test the effects of the program on laptop ownership according to one of our causal identification designs (DD or DDD) directly. However, it still allows us to get a sense of whether students studying in class 12 of government schools in Tamil Nadu were more likely to own laptops in the post-policy period. For this, we exploit variation at the Cohort (Eligible vs Ineligible) and State level (Tamil Nadu vs Other States), similar to our triple difference identification strategy. The results for this exercise are presented in Table 14. As we can observe, households with students studying in Class 12 of government schools in Tamil Nadu were much more likely to own a laptop when compared to other groups.

**Table 14:** Impact on laptop ownership: IHDS 2.

	HH owns laptop
	(1)
*Eligible* × *Treated*	0.022***
	(0.006)
*Eligible*	0.004
	(0.005)
*Treated*	–
	–
*R*^2^	0.03
Observations	1876
Controls	Yes
Year Fixed Effects	Yes
State Fixed Effects	Yes

The table shows results for the impact of TFLS on laptop ownership using the IHDS dataset in the post-policy period. Control variables included as part of different specifications include age, gender, religion, caste, whether any member of the household is literate, highest adult education in the household, number of household members, income quintile of the household, place of residence (rural or urban), percentage of households in the village that have electricity, availability of bank in the village, and availability of a primary school, a middle school, a secondary school, and a private school in the village. Robust standard errors clustered at the state level are reported in parentheses.

*p < 0.1, **p < 0.05, ***p < 0.01.

Having some analytical evidence for higher laptop ownership amongst students impacted by the program, we now examine how this improved access translates into improved foundational math skills for students using our triple difference identification strategy. A potentially direct channel through which this can happen is through access to better educational resources and learning materials for students on account of laptops. If that happens, students will be able to work on their math ability, leading to better foundational skills. Apart from this direct effect, improved math skills of students can also result from two other potential channels: (1) better language comprehension and (2) improved first-stage education outcomes.

### Impact on language comprehension and understanding

Existing literature in the education domain suggests that language proficiency can have potential impacts on math skills of students (Abedi and Lord, 2001; Beal et al., 2010; Brown et al., 2011; Henry et al., 2014; Kieffer et al., 2009; Martiniello, 2008; Staley, 2005).^27^ This is primarily because students might find it difficult to understand the meaning of words mentioned in textbooks as well as those used in math problems. If this happens, then students who lack necessary language skills might suffer from poor math skills as well. In the context of our study, too, it is plausible that improvement in foundational math proficiency is driven by improvement in language skills of the students. This can happen if students are able to understand the meaning of words and sentences better on account of access to better learning resources on laptops.

In the context of Tamil Nadu, the Tamil or English languages are primarily used as a medium of instruction (NUEPA, 2013a, 2013b).^28^ Considering this, the laptops provided under the program had fonts supporting both Tamil and English languages.^29^^,^^30^ While ASER does not provide us with information on the comprehension skills of students in the Tamil Language, it does have information on comprehension skills in the English language. We, therefore, use this information to test the impact of access to laptops on the comprehension skills of students in the English language. We do this by running regressions on a dummy variable that takes the value 1 if students understand the meaning of an English word or sentence and zero, otherwise. Table 15 reports the results for this exercise. As can be observed, students were now more likely to understand and comprehend the meaning of English words on account of access to laptops, which might be a potential reason behind their improved foundational skills in maths. Though a potential concern here can be that the free laptop program was rolled out only in government or government-aided schools, which are much less likely to follow English as the primary medium of instruction when compared to the native Tamil language. Even in that case, English does play an important role as a second language in the context of Tamil Nadu and is taught compulsorily as a subject as well (MHRD, 2014). With most of the learning content available on the internet being in the English language, improved understanding and comprehension of the same can potentially result in improved math skills.

**Table 15:** Potential channel: impact on comprehension of the English language (DDD).

	Meaning	
	English words	English sentences
	(1)	(2)
*Eligible* × *Treated* × *Post*	0.333***	0.019
	(0.059)	(0.020)
*Eligible* × *Treated*	−0.249***	0.006
	(0.046)	(0.019)
*Treated* × *Post*	0.092	0.039**
	(0.056)	(0.018)
*Eligible* × *Post*	−0.074*	0.013
	(0.061)	(0.019)
*Eligible*	0.130**	0.025
	(0.047)	(0.020)
*Post*	−0.095*	−0.125***
	(0.050)	(0.019)
*Treated*	–	–
	–	–
*R*^2^	0.06	0.05
Observations	1340	10,678
Controls	Yes	Yes
Year Fixed Effects	Yes	Yes
State Fixed Effects	Yes	Yes

The table shows results for the impact of TFLS using the ASER dataset. Control variables included as part of different specifications include age, gender, mother’s schooling status, mother’s age, number of household members, electricity connection in the household, electricity in the household on the day of interview (observed use), type of household, electricity in the village, bank in the village, and availability of a primary school, a middle school, a secondary school, and a private school in the village. Robust standard errors clustered at the state level are reported in parentheses.

*p < 0.1, **p < 0.05, ***p < 0.01.

### Impact on first-stage education outcomes

In the education literature, subject-specific skills or test scores are typically considered as “final” educational outcomes (Chatterjee and Poddar, 2021; Fiszbein and Schady, 2009). This is primarily because most of the time improvements in educational learning outcomes are preceded by improvements in “primary” educational outcomes such as number of hours spent studying or students’ school attendance. We test the potential impacts of access to laptops on “primary” educational outcomes such as time spent in school, in private tuition, on homework, as well as school absenteeism in Table 16. As we can observe, students now spend more time in school as well as on doing homework, with significantly fewer hours devoted to private tuition. These effects are likely to be driven in part by high student motivation on account of access to laptops. With the quality of private tuition likely to remain low in developing countries, high student motivation enables students to substitute away from costly private tuition to relatively higher quality self/school learning on account of access to laptops (Bulman and Fairlie, 2016). The results imply that potential positive impacts on education-related behavior and motivation of students in terms of time spent studying in school or on self-study, substituting away from private external (after-school) tuition, might be channels through which laptops can result in improved math skills.

**Table 16:** Impact on educational outcomes.

	IHDS			
	School	Homework	Absent	Pvt. Tuition
	Hours/Week	Hours/Week	Days/Month	Hours/Week
	(1)	(2)	(3)	(4)
*Eligible* × *Treated* × *Post*	8.956***	1.958**	0.255	−2.863***
	(1.598)	(0.753)	(0.942)	(0.836)
*Eligible* × *Treated*	−3.025**	0.615	−0.606	1.574*
	(1.157)	(0.934)	(0.823)	(0.859)
*Treated* × *Post*	4.537***	3.127*	−0.891	−0.281
	(1.163)	(1.580)	(0.714)	(0.437)
*Eligible* × *Post*	0.196	0.057	−0.774	−0.626
	(1.315)	(0.733)	(0.776)	(0.489)
*Eligible*	0.562	0.805	0.126	0.825
	(0.883)	(0.985)	(0.620)	(0.529)
*Post*	1.574	−0.291	1.335	0.966*
	(1.339)	(0.503)	(0.996)	(0.502)
*Treated*	–	–	–	–
*R*^2^	0.12	0.15	0.15	0.17
Observations	2507	2514	2496	2429
Controls	Yes	Yes	Yes	Yes
Year Fixed Effects	Yes	Yes	Yes	Yes
State Fixed Effects	Yes	Yes	Yes	Yes

The table shows results for the impact of TFLS using the IHDS dataset. Control variables included as part of different specifications include age, gender, religion, caste, whether any member of the household is literate, highest adult education in the household, number of household members, income quintile of the household, place of residence (rural or urban), percentage of households in the village that have electricity, availability of bank in the village, and availability of a primary school, a middle school, a secondary school, and a private school in the village. Robust standard errors clustered at the state level are reported in parentheses.

*p < 0.1, **p < 0.05, ***p < 0.01.

## Conclusion and discussion

This study provides empirical evidence for the positive impacts of laptops on the foundational math proficiency of students in the context of India. Using a double difference and a triple difference identification design, we find that the foundational math proficiency of students potentially exposed to the free laptop program improved, with the largest improvements experienced by students in economically disadvantaged households. Furthermore, we find that these positive effects are driven by channels including education-related behavior and motivation of students, as well as better comprehension of the English language. Specifically, we find that students exposed to the program spent more time learning in school and on self-study, coupled with less time spent in private tuition. Students exposed to the program were also more likely to understand the meaning of English words. Our results also suggest larger positive effects of the program for boys when compared to girls, who experience smaller impacts. This can potentially be attributed to a combination of factors, including boys catching up with girls on account of them starting from a lower base, and possible differential selling-off of laptops provided to girls in the open market by economically constrained households. We also find that the program enabled students from poor economic backgrounds to catch up with students from relatively well-off economic status. The paper shows that access to technology in the form of laptops can help improve educational outcomes in developing countries that are likely to lack quality educational infrastructure.

While our study points to positive impacts, how should we think of the efficiency of this policy? The provision of free laptops, as was implemented in the TFLS, entails a high fixed cost. For instance, in the first 3 years of the program, the state government spent over $600 million to distribute over 2 million laptops to students. This implies a cost of roughly $300 per student to achieve a 2.3% increase in foundational math skills. At the outset, these gains from the program in terms of improvement in learning outcomes per dollar spent, therefore, are much smaller when compared to some of the other programs tested in India that were aimed at improving learning outcomes of children. These include, for example, the remedial education program highlighted in Banerjee et al. (2007) or the recruitment of contract teachers intervention analyzed in Muralidharan and Sundararaman (2013).

However, we suggest that a few nuances should be taken into account before deeming the laptop intervention a “*cost inefficient*” way of improving learning outcomes. First, in the paper, we analyze the impacts on foundational math skills of students, which is essentially a below-grade-level outcome or a floor test of math skills. The fact that access to laptops can improve foundational mathematics skills of class 12th students shows that the policy helps the students who are lagging behind the most to catch up. It is plausible that the policy may have even larger impacts for other outcomes, including grade-specific ones. While this is something that we would ideally want to check, the available data in ASER (and IHDS) are limited in this respect. Second, it is important to consider that the policy changed the educational behavior of students in terms of more time spent in school and on self-study, and a shift away from private tuition. This not only results in important cost savings on private tuition for students from economically disadvantaged backgrounds but also inculcates longer-term positive learning habits. Third, the provision of laptops serves as a one-off lump sum transfer to the students as well as households to which these students belong. While the nature of the data and the identification design limit our ability to follow longer-term outcomes and those across multiple domains, this one-off lump sum transfer when provided to students in the form of a laptop, helps to serve them for the long term and is not exhausted in the short term. Hence, ideally, the cost as well as the benefits of the laptop and associated program need to be spread across multiple years in order to think about the efficiency trade-offs involved. Even then, the laptops are likely to generate benefits outside the educational domain, by providing students new opportunities, e.g., through resources for job search or social connections, health information, with implications for other life course domains. The household nature of the resource may further encourage spillover impacts on other household members. Thus, while our study identifies the impacts and heterogeneity in the program for one set of outcomes and channels, we think this captures floor effects that may have longer-term impacts.

While our paper makes important contributions, we would also like to acknowledge some of the limitations of our study. These stem primarily from the lack of availability of quality student-level longitudinal data on grade-appropriate educational outcomes and test scores in India, particularly for upper secondary students. On account of this, our study focuses on the below-grade-level foundational mathematics skills of upper secondary students for its analysis. While estimates from the analysis are important and meaningful from a policy perspective, the incorporation of grade-appropriate test scores could have improved the scope and generalizability of the study. Another potential limitation stemming from data unavailability is that we are not able to perform analysis for students studying in undergraduate degree programs who were potentially exposed to the policy. These limitations, however, serve as potential directions for future research in the field of education in LMICs.

## Supplementary information

Online Appendix: Access to Technology and Foundational Math Proficiency among Students: Empirical Evidence from India

## Data Availability

The paper uses data from the Annual Status of Education Report (ASER) Survey (2008–2014) and India Human Development Survey (IHDS), Round 1 and 2 (2004–2005, 2011–2012) for the purpose of analysis. More specifically, we use the following files: (1) Household files from ASER (2008–2014), (2) Individual files from IHDS (2004–2005, 2011–2012), (3)Village files from IHDS (2004–2005, 2011–2012). The ASER dataset can be accessed by writing to the ASER team at contact@asercentre.org. Data for the IHDS individual panel (2004–2005, 2011–2012) can be downloaded from: https://www.icpsr.umich.edu/web/DSDR/studies/37382; Data for the IHDS village file (2004–05) can be downloaded from: https://www.icpsr.umich.edu/web/DSDR/studies/22626; Data for the IHDS village file (2011–12) can be downloaded from: https://www.icpsr.umich.edu/web/DSDR/studies/36151. All the codes used for estimating final results are made available via an OSF repository for the project at https://osf.io/2hspx/?view_only=1907ffb22bd2474e8cc6247013e959b4.

## Footnotes

1 See: sdgs.un.org/goals/goal4.

2 See: sdg4-data.uis.unesco.org/.

3 See: https://www.dtnext.in/news/tamilnadu/free-laptop-scheme-on-sleep-mode-735064.

4 See: laptop.org/aboutolpc.

5 See: www.thehindu.com/news/national/tamil-nadu/who-got-tns-free-laptops/article6999363.ece

6 Government schools and colleges are owned and operated by the central or the state governments. Government-aided institutes, on the other hand, receive funds from the government exchequer.

7 See: johnsonasirservices.org/web/Download3/LAP%20TOP%20COMP.spi_e_17_2011.pdf.

8 See: www.ndtv.com/assembly/tamil-nadu-polls-aiadmk-offers-laptop-mixies-gold-for-mangalsutra-451017.

9 See: www.economictimes.indiatimes.com/news/politics-and-nation/karunanidhi-promises-free-laptops-to-all-students-on-victory/articleshow/7779043.cms?from=mdr.

10 Some secondary sources also suggest that the laptops included Open Office applications such as Word, Spreadsheets, and PowerPoint. See: www.prayatna.typepad.com/education/2011/10/68-lakh-free-laptops-to-students.html.

11 See: www.techrights.org/wp-content/uploads/2011/08/ICB_LTC_scheme_PI_2_final.pdf.

12 https://cag.gov.in/webroot/uploads/download_audit_report/2022/Report-No-10-of-2022-School-Education-2020-21-English-064427c54a43990.22472310.pdf.

13 See: www.thehindu.com/news/national/tamil-nadu/who-got-tns-free-laptops/article6999363.ece.

14 See: www.indiatoday.in/india/story/jayalalitha-tamil-nadu-free-laptop-scheme-students-aiadmk-free-colour-tv-scheme-158384-2013-04-09.

15 See: https://www.deccanchronicle.com/140905/nation-current-affairs/article/free-laptops-improve-tech-skills-tamil-nadu-students-survey?infinitescroll=1.

16 See: riseprogramme.org/blog/shifting-schooling-learning-aser-shaped-indias-education-discourse-policy-measuring-learning#:~:text=ASER%20is%20now%20the%20largest,%2C%20state%2C%20and%20national%20levels.

17 See: asercentre.org/wp-content/uploads/2022/12/2.-frequentlyaskedquestionsaboutaser-2.pdf.

18 Classified as state at the time of the survey.

19 See: asercentre.org/wp-content/uploads/2022/12/2.-frequentlyaskedquestionsaboutaser-2.pdf.

20 Students residing in non-durable “Kutcha Houses”.

21 See: indiatoday.in/india/story/jayalalitha-tamil-nadu-free-laptop-scheme-students-aiadmk-free-colour-tv-scheme-158384-2013-04-09.

22 See: livemint.com/Opinion/Jbwccz7xGillDmV5QKL0xL/The-perils-of-populism.html.

23 See: https://www.unicef.org/press-releases/girls-worldwide-lag-behind-boys-mathematics-failed-discrimination-and-gender.

24 We include all the states of India as a control group except Uttar Pradesh, as it had initiated a large-scale laptop policy for school students in the year 2013. https://www.bbc.co.uk/news/world-asia-india-21738237.

25 We also run a robustness check in Appendix Table A.1, where we only use southern states of India as a control group and find similar estimates.

26 See: https://www.education.gov.in/sites/upload_files/mhrd/files/statistics-new/SISH201112.pdf.

27 See:kennedykrieger.org/stories/linking-research-classrooms-blog/how-do-language-skills-impact-math-learning.

28 See: www.education.gov.in/sites/upload_files/mhrd/files/statistics-new/SISH201112.pdf.

29 See: techrights.org/wp-content/uploads/2011/08/ICB_LTC_scheme_PI_2_final.pdf.

30 See: cms.tn.gov.in/sites/default/files/documents/spi_e_pn_2019_20.pdf.
