# Supplementary material for: Access to technology and foundational math proficiency among students: empirical evidence from India
Source: Humanit Soc Sci Commun. 2025 Jul 4;12(1):1015. doi: 10.1057/s41599-025-05224-w (PMC12227312; doi:10.1057/s41599-025-05224-w)
Supplement: Supplementary file 1 — Online Appendix: Access to Technology and Foundational Math Proficiency among Students: Empirical Evidence from India [file 41599_2025_5224_MOESM1_ESM.pdf]

Online Appendix: Access to Technology and Foundational Math Proficiency among Students: Empirical Evidence from India

A Appendix

Figure A.1: Map of India

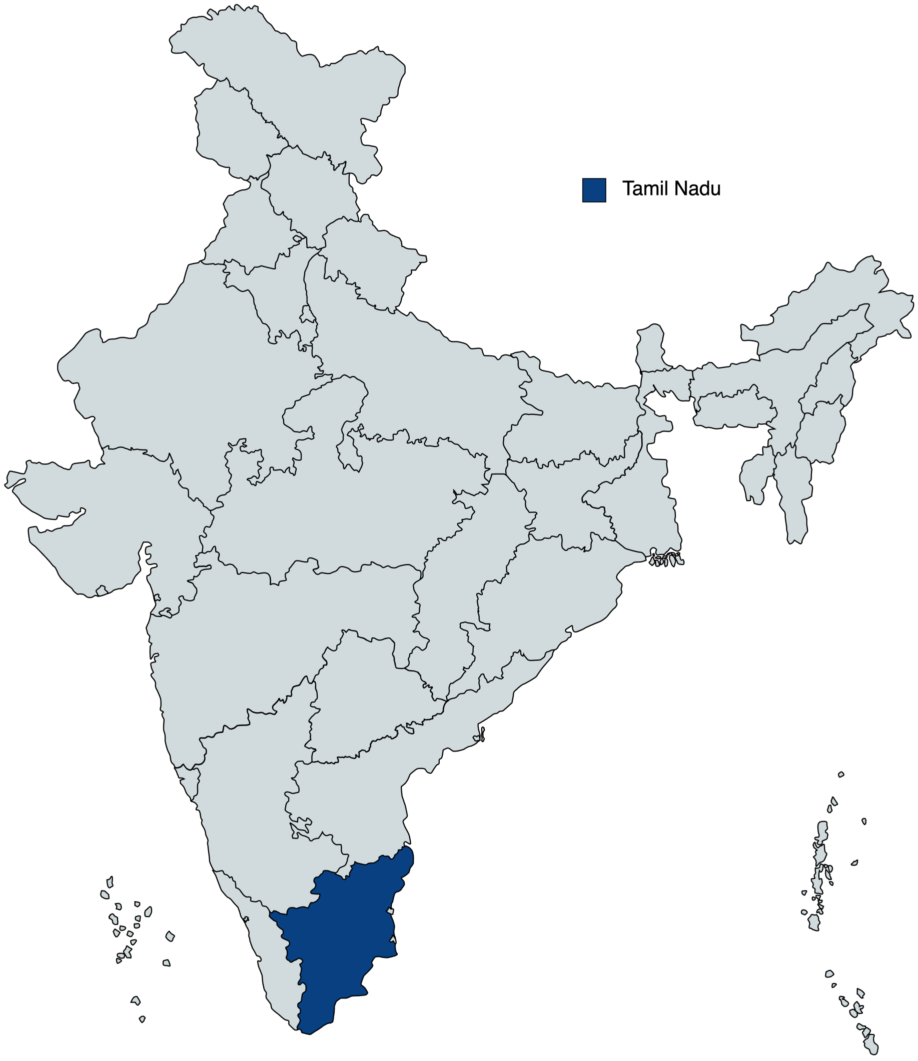

Figure A.2: Laptop distribution under Tamil Nadu Free Laptop Scheme

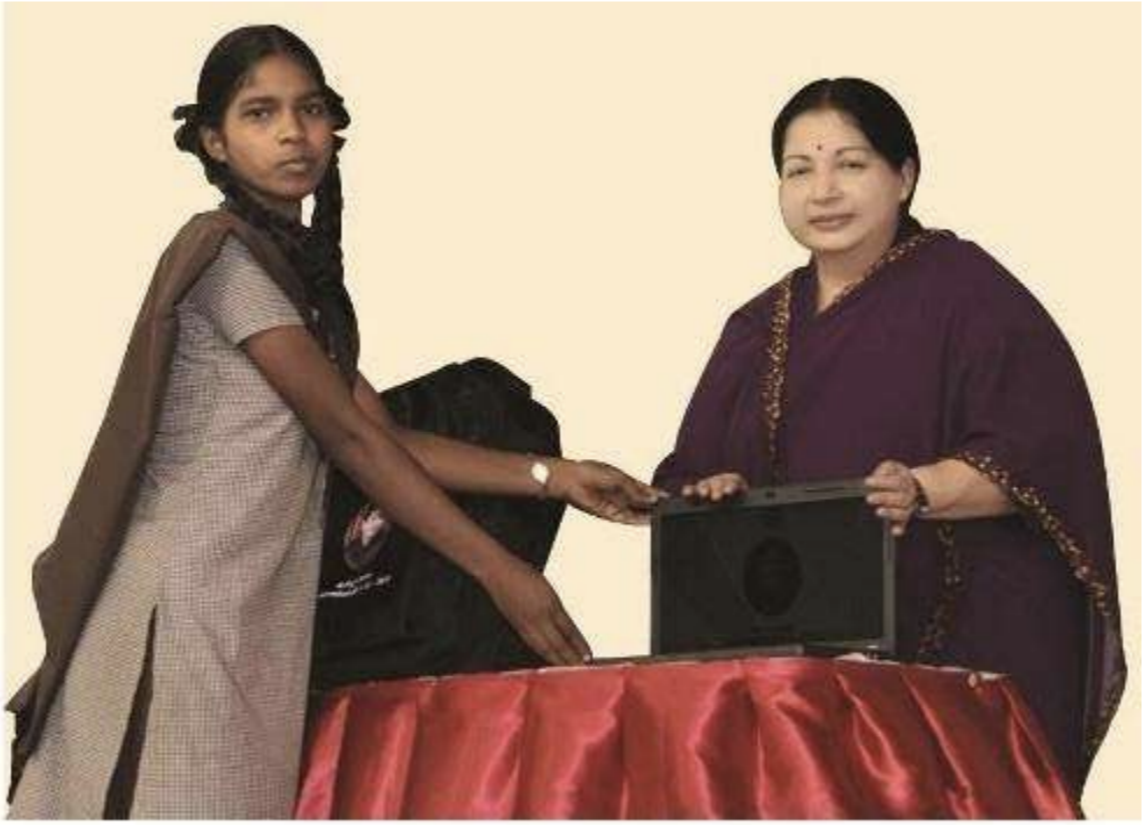

Source: Government Report (See: cms.tn.gov.in/sites/default/files/documents/spi_e_pn_2019_20.pdf)

Figure A.3: Laptop distribution under Tamil Nadu Free Laptop Scheme

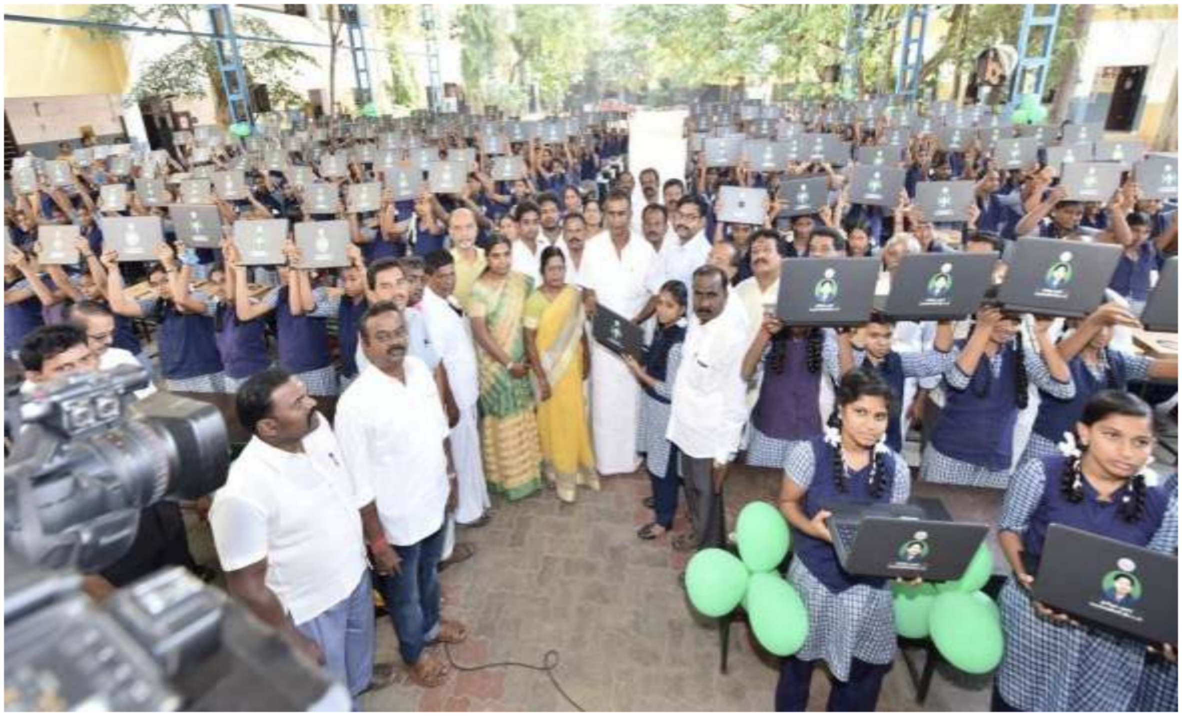

Source: Government Report (See: cms.tn.gov.in/sites/default/files/documents/spi_e_pn_2019_20.pdf)

Figure A.4: Sample Test Exercises in Math

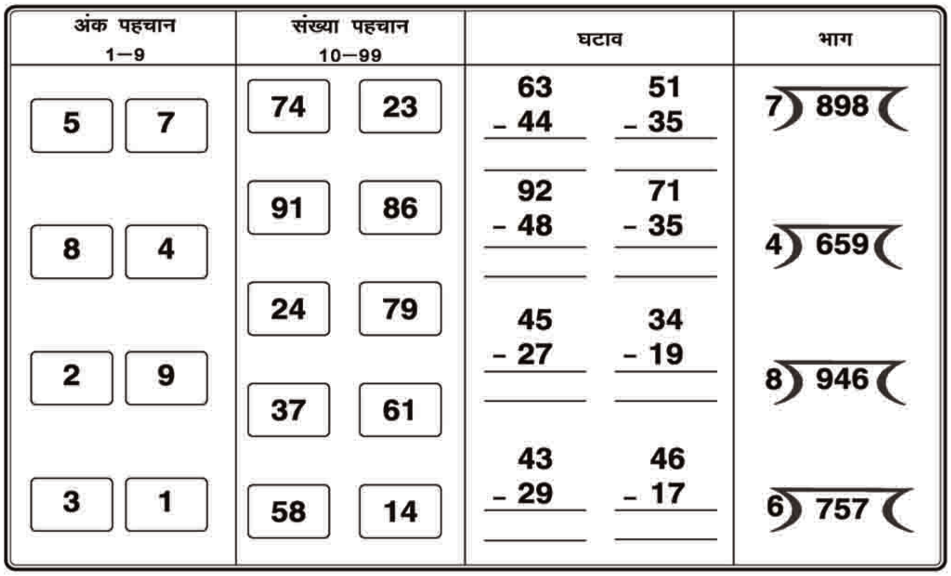

अंक पहचान 1–9		संख्या पहचान 10–99		घटाव		भाग
5	7	74	23	63 − 44	51 − 35	7) 898
8	4	91	86	92 − 48	71 − 35	4) 659
		24	79	45 − 27	34 − 19	
2	9	37	61			8) 946
3	1	58	14	43 − 29	46 − 17	6) 757

Table A.1: Impact on Math Score: Only Southern States as Control Group

	(1)
Eligible × *Treated* × *Post*	0.077*
	(0.028)
Eligible × *Treated*	-0.028
	(0.014)
Treated × *Post*	-0.113**
	(0.027)
Eligible × *Post*	0.006
	(0.027)
Eligible	0.028
	(0.016)
Post	-0.348***
	(0.025)
Treated	-
	-
R^2	0.09
Observations	8,116
Controls	Yes
Year Fixed Effects	Yes
State Fixed Effects	Yes

Notes: The table shows results for the impact of TFLS using ASER dataset. Control variables included as part of different specifications include age, gender, mother's schooling status, mother's age, number of household members, electricity connection in the household, electricity in the household on the day of interview (observed use), type of household, electricity in the village, bank in the village, and availability of a primary school, a middle school, a secondary school, and a private school in the village. Robust standard errors clustered at the state level are reported in parentheses. *** p<0.01 **p<0.05 *p<0.1
